# Drug Toxicity Evaluation Based on Organ-on-a-Chip Technology: A Review

**DOI:** 10.3390/mi11040381

**Published:** 2020-04-03

**Authors:** Ye Cong, Xiahe Han, Youping Wang, Zongzheng Chen, Yao Lu, Tingjiao Liu, Zhengzhi Wu, Yu Jin, Yong Luo, Xiuli Zhang

**Affiliations:** 1 State Key Laboratory of Fine Chemicals, Department of Chemical Engineering, Dalian University of Technology, Dalian 116023, China; cyciel96@163.com; 2 College of Pharmaceutical Science, Soochow University, Suzhou 215123, China; 20194226017@stu.suda.edu.cn (X.H.); 20194226030@stu.suda.edu.cn (Y.W.); 3 Health Science Center, Shenzhen University, Shenzhen 518060, China; chenmond@foxmail.com (Z.C.); szwzz001@email.szu.edu.cn (Z.W.); nnjinyu@sina.com (Y.J.); 4 Biotechnologhy Division, Dalian Institute of Chemical Physics, Chinese Academy of Sciences, Dalian 116023, China; luyao@dicp.ac.cn; 5 College of Stomatology, Dalian Medical University, Dalian 116011, China; tingjiao@dmu.edu.cn

**Keywords:** organ-on-a-chip, drug toxicity, drug metabolism

## Abstract

Organ-on-a-chip academic research is in its blossom. Drug toxicity evaluation is a promising area in which organ-on-a-chip technology can apply. A unique advantage of organ-on-a-chip is the ability to integrate drug metabolism and drug toxic processes in a single device, which facilitates evaluation of toxicity of drug metabolites. Human organ-on-a-chip has been fabricated and used to assess drug toxicity with data correlation with the clinical trial. In this review, we introduced the microfluidic chip models of liver, kidney, heart, nerve, and other organs and multiple organs, highlighting the application of these models in drug toxicity detection. Some biomarkers of toxic injury that have been used in organ chip platforms or have potential for use on organ chip platforms are summarized. Finally, we discussed the goals and future directions for drug toxicity evaluation based on organ-on-a-chip technology.

## 1. Introduction

Animal models have been widely used in the field of pre-clinical testing and toxicity testing of drugs [1]. However, the results obtained by animal models are often different from those of humans, which has led to the withdrawal of many drugs from the clinical stage due to adverse effects. Traditional in vitro 2D cultures can replace animal models to a certain extent, but cannot replicate the physiological environment of cells in vivo. Moreover, the lack of cell-to-cell and cell-to-matrix interactions in 2D cultures often leads to the loss of cell function. Therefore, building more bionic and stable in vitro research models has gradually become a hot topic.

Microfluidic chip development is a branch of technology that studies micro/sub-micron scale fluid control. Initially, microfluidic chips were used to replace the traditional analytical method of laboratory analysis by constructing a “µ-TAS” or “lab-on-a-chip” model with microchromatography and capillary electrophoresis [2]. With the widespread application of microfluidic chip technology, people began to apply it to the fields of cell biology and cell analysis, and technically realized cell culture on-chip, simulation and construction of in vivo microenvironment on-chip, single-cell analysis chips, and further “organ-on-a-chip” models. [3,4].

Organ-on-a-chip is a specialized subtype of the microfluidic chip which mimics functional units of human organs in vitro. It includes the cell microenvironment, and the cell-to-cell co-culture and interactions. The organ-on-a-chip model can more accurately adjust the environmental conditions related to its growth and function according to the type of cells it cultures. Flow culture can control the supply of nutrients and remove accumulated cellular waste or secondary metabolites from the culture medium. Also, it can regulate the oxygenation level, shear stress guarantees the barrier integrity of the cell layer, and it can control cell migration in vitro [5]. On the organ-on-a-chip models, one can use cells to regenerate many functions that are difficult to achieve in traditional in vitro models. For example, reconstruction of tissue barrier function, cultivation of parenchymal tissue, and integration of multiple organ functions [6]. In the last decade, academic research on organ-on-a-chip systems has boomed. Researchers have also achieved further development of organ-on-a-chip technology.

Cell-based 3D culture models spheroids and organoids are widely used in in vitro tissue and organ reconstruction. Spheroids can be used to reproduce tissues or organs such as tumors, the liver, and nerves in vitro from primary cells or cell lines [7,8,9]. Organoids are a new three-dimensional culture model enabling miniaturized organs produced by stem cells in vitro. Through appropriate growth factor treatment, stem cell differentiation and self-organization into organ-specific cell types and tissues occurs, recreating the structure and function of organs in vitro [10]. Combining the 3D culture of organs or tissues with microfluidic chip technology can provide a more suitable differentiation and culture environment and improve tissue or organ function and sensitivity to stimulation. Therefore, people began to use spheroid/organoid-on-a-chip for physiological in vitro research. Some of the latest applications have been described by Dongeun Huh [11]. The article pointed out that this technology was expected to be the most advantageous weapon to solve the key issues of new drug development. Furthermore, compared with the traditional organoid technology, the organ chip can more accurately control the local environment and simulate human organ function in vitro, which is more advantageous in applications.

Drug toxicity is one of the major reasons for post-market drug withdrawal; therefore it is crucial to perform preclinical toxicity testing on candidate drugs. Owing to the existence of species differences, the results obtained from animal models cannot be fully adapted to humans [12]. In order to improve the accuracy of drug preclinical test results, many in vitro models have been constructed for toxicity screening, and organ-on-a-chip is a superior one.

To simulate the toxic effects of drug candidates on different cells, tissues, or organs of the human body, scientists have constructed many organ-on-a-chip models that simulate single or multiple organs, e.g., the liver [13]-, kidney [14]-, heart [15]-, nerves [16]-, and multi-organs-on-chips [17]. These models have been proven to have the potential to develop into new platforms for drug toxicity detection [18]. In this review, we will summarize the latest drug toxicity assessments based on organ chips.

## 2. Biomarkers for Drug Toxicity Testing

The selection of appropriate biomarkers is critical for drug toxicity testing, and the high expression of biomarkers in in vitro models will lower the detection limit. This section summarizes the toxic biomarkers used in in vitro models (Table 1).

## 3. Drug Hepatotoxicity Testing by Liver-on-a-Chip

### 3.1. Overview of Liver and Hepatotoxicity

The liver is the largest internal organ in the human body. Its main function is metabolism, and it also plays a central role in detoxification, storage of glycogen, and synthesis of secreted proteins in the body. Liver is a major target organ of drug toxicity. Drug-induced liver injury (DILI) can cause drug withdrawal from market and acute and chronic liver disease, and is a major safety issue in drug development. Liver-on-a-chip is a state-of-art technique to detect drug hepatotoxicity.

### 3.2. Research Progress of Liver Chips for Drug Toxicity Testing

Before inception of the liver chip, preclinical determination of drug hepatotoxicity relied on immortalized cell lines and primary isolated hepatocytes (HepG2) [60]. Rapid loss of activity and reduced liver specific functions and gene expression are common shortcomings of these models [61]. Animal experiments have long experimental cycles, are costly, and have ethical and animal welfare problems. Therefore, there is a need to establish a new in vitro platform that can preserve the cell microenvironment in vivo, simulate various functions of liver tissues in the body, and achieve long-term repeated administration. Liver chips meet these requirements.

Bhise et al. developed a liver chip for long-term culture of human HepG2 cells or C3A cells. HepG2 cells or C3A cells were embedded in photo-crosslinked gelatin methacrylate hydrogel to make spheroids. The spheroids were arranged in a microfluidic chip and liver toxicity was assessed by detecting changes in specific markers. In this platform, the spheroids still retain their activity for 30 days, and have proven their potential for drug toxicity assessment [62]. Zuchowska et al. [63] also used 3D spherical culture of HepG2 cells to detect the hepatotoxicity of the anti-cancer drug 5-fluorouracil (5-FU), and found that as the diameter of the sphere increased, HepG2 cells reduced resistance to 5-FU at two test concentrations.

**Figure 1:**
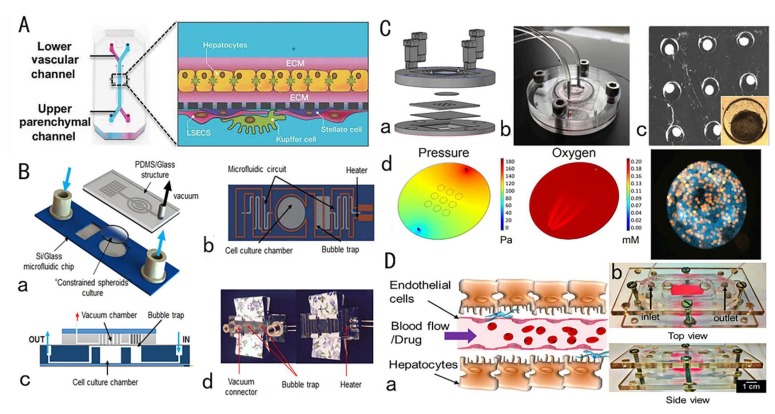
A liver chip system for drug toxicity testing. (**A**) Specific cell type (human, dog, rat) four-cell culture chip used for drug toxicity test [66]; (**B**) Perfusion-incubator-liver-chip (PIC) using CO_2_ gas pressure to drive the perfusion medium, which can be used for chronic toxicity repeated dose drug toxicity tests [67] (**C**) Liver chip combined with fluorescent probe to track mitochondrial function status in real time to monitor cell viability and metabolic function [68]; (**D**) Liver chip microdevice with a vascular layer, which is helpful for predicting the mechanism of drug liver toxicity at the microcirculation level [69].

Besides the spheroidal culture, co-culture techniques also have been widely used in liver chips to establish a microenvironment that is closer to the body and maintains liver cell functions. Especially, co-culture of parenchymal cells and non-parenchymal cells (stellate cells, sinusoidal endothelial cells, Kupffer cells) is important for achieving cell-to-cell contact and transmission of signal molecules, enhancing liver cell functions. Kostadinova et al. [64] established a nylon scaffold that allows three-dimensional culture of HepG2 and non-parenchymal cells including Kupffer cells, stellate cells, sinusoidal endothelial cells, and bile duct endothelial cells, which can maintain the specific functions of the liver long-term, form bile duct-like structures, and respond to inflammatory stimuli. Ma et al. [65] developed a microfluidic device that mimics the hepatic lobular-like structure. The experiments found that co-culture of HepG2 and non-parenchymal cells (HAECs) formed hepatic cord-like structures and hepatic sinusoidal structures. The ability to maintain co-culture of HepG2 and non-parenchymal cells makes this system an alternative for hepatotoxicity testing. This study proved that the combination of three-dimensional culture and co-culture in the liver chip can better mimic the complex human liver system, which not only achieved longer-term culture and maintained good liver function, but also simulated the toxic response of whole liver organ to drug candidates. Jang et al. [66] constructed a human, dog, and rat two-cell and four-cell species-specific liver chip, and used this chip in combination with microscopic observation, fluorescent staining, and biomarker labeling to verify a variety of phenotypes for drug-induced liver injury, correlation between species-specific liver chips and human liver chips, and identification of specific drug-induced liver toxicity damage and mechanisms (Figure 1A).

When culturing the hepatic cells in liver chips, perfusion culture technology has been widely employed. Deng et al. [70] constructed a hepatic sinusoidal chip device consisting of four transformed cell lines and perfused it with a peristaltic pump. The cell composition, proportion, and spatial arrangement were similar to the physiological characteristics of hepatic sinusoids in the body, enhancing the sensitivity of HepG2 cell toxicity drug testing, and also realizing the study of drug–drug interactions. In addition to peristaltic perfusion, gravity-driven, paper-based siphon and CO_2_ gas-driven perfusion methods were also effectively applied to liver chips. Yu et al. [67] constructed a perfusion-incubator-liver-chip (PIC) that integrated air bubbles and temperature processing functions. In addition, it drove the flow using CO_2_ pressure and achieved pH control, maintaining liver cell viability for 14 days with persistent good function. This chip was used to investigate acute and chronic drug hepatotoxicity of acetaminophen and diclofenac (Figure 1B). Jang et al. [71] added a phase guide to a 3D perfusion chip without using a pump. In recent years, innovations in perfusion culture technology have not only enabled better simulation of the in vivo microenvironment, but also simplified chip fabrication, making it more suitable for drug toxicity testing.

The occurrence and development of liver injury is a dynamic process and cannot be correctly characterized using only endpoint measurements. In this regard, by combining the liver chip with other biosensors, real-time monitoring of many cellular parameters can be achieved. Bavli et al. proposed a liver chip device that tracks the dynamics of mitochondrial dysfunction and simulates liver damage in real time. Phosphorescent microprobes are embedded in the liver chip, and continuous electrochemical measurements of glucose and lactose changes are achieved through computer-controlled microfluidic switches. Changes in mitochondria from oxidative phosphorylation to anaerobic glycolysis indicate impaired mitochondrial function to assess changes in cell viability and metabolic function [68] (Figure 1C). In addition, some liver chips have also developed high-throughput rapid monitoring systems to quickly evaluate the efficacy and toxicity of drugs. Riahi et al. [72] developed a microfluidic bead electrochemical immunosensor for long-term continuous measurement of cell-secreted biomarkers, which was used in combination with the liver chip to achieve online monitoring of cell-secreted biomarkers, which not only achieved measurement accuracy but also greatly improved detection efficiency.

The miniaturization and flow-through operation of the microfluidic chip greatly reduces sample consumption, and significantly improves detection reliability, resolution, and sensitivity. Kwon et al. [73] designed a Team chip that can predict liver toxicity based on metabolism. Liver chips that can be used for high-throughput screening not only simplify data collection, but also reduce research costs. In addition, the combination of 3D bioprinting technology and organ chips is a promising research direction. Massa et al. used GelMA hydrogel embedded with HepG2/C3A cells as a scaffold, implanted human umbilical vein endothelial cells (HUVECs) into hollow microchannels to construct a vascular layer, and combined this with 3D bioprinting technology, and developed a perfusion liver chip with a vascular layer. The biomimeticity of the chip was enhanced, and the effect of the endothelial layer on the APAP administration process was simulated. The device is useful for observing and predicting the mechanism of drug circulation toxicity at the microcirculation level [69] (Figure 1D). In addition, Nguyen et al. [74] combined patient-derived primary liver cells with 3D bioprinting technology to effectively simulate Drug-induced liver injury (DILI). Using 3D bioprinting technology to achieve rapid high-throughput production of liver chips or liver tissue will greatly reduce production costs and reduce operational difficulties.

Very recently, the formation of organoids from stem cells has emerged as a focus of research. Combining organoid cultivation with organ chips can better control the organoids and their growth microenvironment, and strengthen the interaction between tissues and multiple organs. The research in this area is still ongoing [75].

### 3.3. Brief Summary

Advanced techniques, including spheroidal culture, co-culture, perfusion, and 3D bioprinting, have been used in liver chip research, and made liver chip mimics more and more realistic. Organoids combined with liver chips show great potential and are the future research directions. In the field of hepatotoxicity assessment, the liver chip is promising owing to the advantages of increased accuracy, sensitivity, and the ability to detect dynamics. Liver chip technologies are especially important for China because DILI frequently occurs in China due to wide use of health products and Chinese medicine [76].

## 4. Drug Nephrotoxicity Testing by Kidney-on-a-Chip

### 4.1. Overview of Drug-Induced Nephrotoxicity

Drug-induced nephrotoxicity (DIN) is a poisonous effect of drugs on renal function. In clinical settings, DIN accounts for about ~25% of the reported severe adverse drug reactions [1]. At present, animal and cell models are the common tools used to detect DIN. Owing to the species differences between animals and humans, and the accuracy of the results of renal toxicity prediction, using animal models is inferior. Cell models often utilize simple 2D cultures and have low sensitivity to many in vivo biomarkers. Recently, the kidney-on-a-chip model has been utilized to test nephrotoxicity, which can realize the co-culture of a variety of cells, the simulation of the in vivo microenvironment, and in vitro biomarker profiling. This type of model has great potential to be developed as a future standard in DIN screening.

### 4.2. Kidney-on-a-Chip for Drug Nephrotoxicity Testing

**Figure 2:**
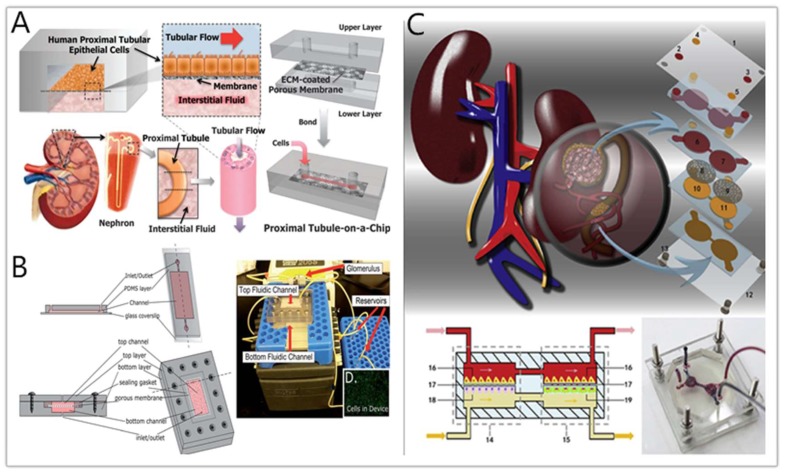
A kidney chip system for drug toxicity testing. (**A**) Design of the human kidney proximal tubule-on-a-chip [77]. (**B**) Microfluidic device designs for single channel and multi-channel devices, and the final device assembly with notable features marked [81]. (**C**) Design for the device contains the glomerulus, Bowman’s capsule, proximal renal lumen, and capillary tubules [86].

A common type of kidney-on-a-chip is to culture the renal cells on extracellular matrix (ECM)-coated membranes in a sandwiched structure between the polydimethylsiloxane (PDMS) layer and the microfluidic channel [77,78,79]. The LeClerc group [78] established their microfluidic kidney chips to study acrolein and ifosfamide nephrotoxicity. Van der Meer’s team [38] have integrated TEER (transendothelial resistance) measurements in these platforms to assess the cell barrier functions of the related cells, such as human renal epithelial and dog renal tubular cells. Jang’s group [77] measured the cisplatin toxicity and P-Glycoprotein (P-gp) efflux transporter activity on a human proximal tubule chip. Compared with traditional cell models, the results obtained on the chip model are closer to in vivo experiments. This is the first reported nephrotoxicity study using human primary renal tubular cells on an “organ-on-a-chip” platform (Figure 2A). Kim et al. [80] cultured Madin-Darby canine kidney (MDCK) cells on a fibronection-coated porous membrane to construct a sandwich structured PDMS chip and used the model to study the kinetics of gentamicin nephrotoxicity. Through the detection of KIM-1, cell death, ZO-1 expression, and other indicators, it was concluded that high concentration exposure to gentamicin would destroy the tight junctions between MDCK cells, reduce cell viability, and increase membrane permeability, which demonstrated the potential of the kidney chip for drug nephrotoxicity research.

Sakolish’s group [81] used immortal cell lines to simulate the function of the glomerulus and proximal tubule (Figure 2B). In addition to providing a shear-stressed culture environment for HK-2 cells, the platform contained a glomerular filter containing HUVECs to provide a more realistic “primary urine” inside the device. Later, the group used such a platform to evaluate the DIN by cisplatin and cyclosporine [82]. The results show that HK-2 cells cultured in this microfluidic device are more sensitive. Musah et al. demonstrated that fluid shear stress could promote the proximal tubular epithelial cells (PTECs) to retain more mature phenotypes and develop a glomerulus-on-a-chip model [82]. Weber and his team [83] combined the single-channel Norvatis microphysiology system (MPS) and the PTECs to reconstruct the physiological function of human proximal tubules in vitro and used this platform to evaluate the nephrotoxicity of polymyxin antibiotics. When PTECs were exposed to polymyxin B, some significant increases in damage signals were observed, including KIM-1 and a group of damage-related miRNAs. The platform can perform safety detection of new compounds and define different toxicological pathway responses of the molecule.

Primary rat kidney cells are also used in current kidney-on-a-chips. Wang’s group [84] constructed a glomerular chip that reconstructed part of the kidney function at the organ level to simulate the glomerular stimulation in the high glucose environment. The device consists of parallel channels, in which primary glomerular microtissues are arranged, including glomerular endothelial cells, three-dimensional basement membranes, and podocytes. These tissues combine with fluid flow to mimic the glomerular filtration barrier (GFB). Qin et al. [85] cultured primary rat glomerular endothelial cells (GECs) on a microfluidic chip to simulate the selective permeability of the renal barrier and then studied the cadmium-induced nephrotoxicity by detecting the expression of ZO-1 protein by GECs. Qu et al. [86] have constructed a microfluidic platform that mimics nephrons, and the device contains glomerulus, Bowman’s capsule, proximal renal lumen, and capillary tubules (Figure 2C). Cisplatin and doxorubicin were added to the “renal blood flow” containing bovine serum albumin, E-cadherin, and vascular endothelial growth factor (VEGF), and other biomarkers were used to record the damage of a variety of primary kidney cells through fluorescence imaging. The results showed that the complex microenvironment built in the device makes kidney cells more sensitive to drug nephrotoxicity, which provided new insights for drug-induced nephrotoxicity detection.

### 4.3. Brief Summary

Kidney-on-a-chip has shown advantages in nephrotoxicity testing. For instance, the presence of fluid shear stress makes cells more sensitive to the drugs, and the use of primary cells makes the in vitro renal toxicity testing more physiologically relevant. However, high-throughput screening on the kidney chip platform has not yet been achieved. Besides, nephrotoxicity induced by drug metabolites can hardly be studied directly by kidney-on-a-chip. Hyphenation of other organ chips, especially liver chips, is desirable for in-depth study of nephrotoxicity of novel drug candidates. There also is an urgent need to systematically combine in vitro, in vivo, and clinical examinations to develop a set of safe biomarkers that can be effectively and safely detected and treated for reliable diagnosis and monitoring of kidney damage and recovery.

## 5. Drug Cardiotoxicity Testing by Heart-on-a-Chip

### 5.1. Overview of Drug-Induced Cardiotoxicity

“Cardiotoxicity” refers to the direct damage to the heart tissue caused by drugs and the indirect damage by the effects of drugs on hemodynamics, such as causing heart thrombosis [87]. “Cardiotoxicity” was first proposed in 1946 to describe the toxic effects of local anesthetics, mercury diuretics, and digitalis on the heart. Later in the 1970s, it was used to describe cardiac complications caused by anthracycline, adriamycin, and 5-fluorouracil [88].

Cardiotoxicity is one of the major reasons of drug failure in clinical testing and withdrawal of drugs from market. For example, drug cardiotoxicity can result in cardiac arrhythmias, therefore the preclinical screening of drug cardiotoxicity is crucial [89]. At present, two-dimensional in vitro cell culture models and animal models are predominant; however, planar cell models cannot restore the complex structure and microenvironment of the heart tissue in the body, and animal models differ from humans in terms of ion channels, biological pathways, and pharmacokinetics. They cannot yet fully reproduce human physiological functions, and in many cases, cannot accurately predict whether drugs have cardiotoxicity to the human body. Other cardiotoxicity screening techniques, such as patch clamps, often involve labor-intensive and invasive cell operations [90]. It is necessary to develop an in vitro model that is more relevant and accurate and simple to operate for cardiac toxicity testing.

### 5.2. Heart-on-a-Chip for Drug-Induced Cardiotoxicity Testing

**Figure 3:**
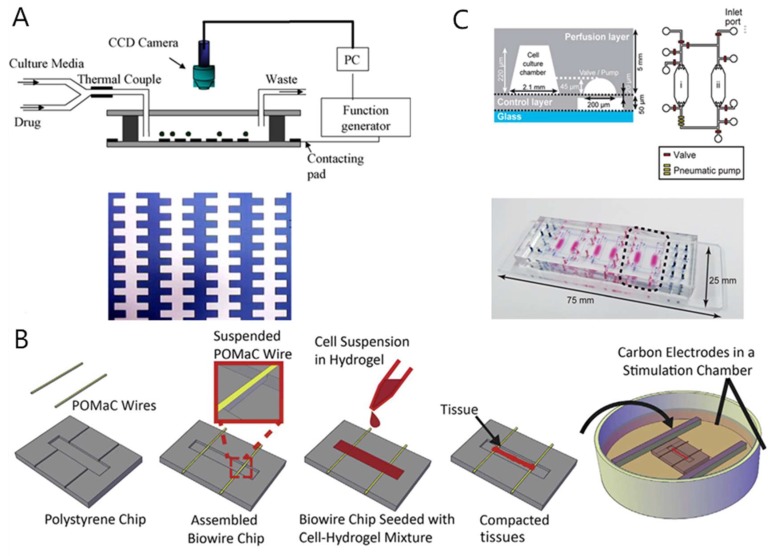
A heart chip system for drug toxicity testing. (**A**) Schematic of the first microfluidic devices to CMs anisotropy [91]. (**B**) Representative tissues in the Biowire II platform [96]. (**C**) Design of the microfluidic device used for integrated heart/cancer-on-a-chip (iHCC) [98].

Electrical stimulation can promote the in vitro maturation of cardiomyocytes. Cardiac chips adopting electrical stimulation have gradually matured. Yang Jianmin et al. [91] reported the first microfluidic devices to achieve cardiac myocytes (CMs) anisotropy (Figure 3A). Qian et al. [92] constructed a cardiac chip that consists of a microelectrode array (MEA) for potential field indication and an interleaved electrode array for conversion. The platform uses transplantation of cardiomyocytes (CMs) derived from human induced pluripotent stem cells (hiPSC-CMs) to measure the electrophysiology and contractility of myocardial cells under physiological conditions and drug stimulation, respectively. The electrical stimulation using microfabricated electrodes (mainly MEA) significantly enhanced the structure and arrangement of cardiac cells. The platform tested the effects of norepinephrine, a clinical drug used to treat hypotension and heart failure. Maoz et al. [47] established a microfluidic device that integrates MEAs and electrodes to verify tissue barrier function by TEER, which helps to understand cardiac pharmacodynamics. The device consisted of an MEA, a PDMS layer, a polyethylene terepthalate (PET) film, a top chip channel, and a TEER electrode. The PET membrane was covered with a layer of HUVECs, and hiPSC-CMs were cultured on the MEA surface. The device can be used to monitor the inflammatory response and the effects of epinephrine drugs on cardiac function. Caluori et al. [93] integrated an electro-mechanical cell biosensor on the heart chip, which could simultaneously detect cardiac excitation–contraction coupling. They used this system to test the response of cells under the stimulation of drugs, and the results show that the system produces a dose-dependent response to calcium channel drugs, isoproterenol, and verapamil, which proves that the device has potential for application in drug screening and drug cardiotoxicity testing. Ville et al. [45] constructed a microelectrode array chip based on human pluripotent stem cell-derived cardiomyocytes and a multi-stage array. This method is capable of constructing layered heart tissue similar to natural myocardium and measuring electrophysiological data. This system proves that hiPSC-CMs has the potential to be used for drug cardiotoxicity screening, by measuring the drug response of hiPSC-CMs in the presence of cardiotoxic prodrugs (terfenadine) and its non-cardiotoxic metabolites (fexofenadine).

In addition to electrical stimulation, there are currently many cardiac chips that attempt to reproduce the annular uniaxial strain in vitro. Marsano et al. [94] developed a cardiac chip with hydrocardial coating, which promoted cardiomyocyte maturation through conductive mechanical stimulation, improved electrical stimulation pacing performance, and enabled analysis of cardiac tissue pacing. The Parker group used muscle thin-film technology (MTF) to construct the heart chip, and further increased the throughput of MTF. This device reflected the effect of isoprenaline, a β-adrenergic receptor agonist, on the contractility of ventricular CMs in neonatal rats [46]. On the basis of this, the Parker group then assembled flexible thin-film sensors and developed a high-throughput microfluidic device, in which the contractile stress and pulsation rate of 24 cardiac muscle tissues could be read at the same time. Recently, a Biowire platform was proposed in which caridac cells were embedded in hydrogels in PDMS microwells, and heart tissue was suspended in holes on two polymer wires, mimicking the growth of muscle fibers in the heart. When they contract, they pull the wires to produce a measurable deflection, which is transformed into a heartbeat signature [95]. In the latest research, Zhao et al. [96] for the first time use a biodegradable, elastic, UV-polymerizable, and rapid-producing poly(octamethylene maleate (anhydride) citrate) (POMaC) polymer material to create a two-dimensional grid for cell growth, called the Biowire II platform. It is a heteropolar cardiac tissue engineered with different atria and ventricles that provides simultaneous quantification of force and Ca^2+^ transients (Figure 3B).

Although the vast majority of cardiac chip devices mimic the myocardium, studies of cardiovascular models and systems that combine the heart and other organ tissues have been reported in recent years. Ellis et al. [97] developed a microfluidic device that mimics human myocardium and its surrounding microvasculature by spatially controlling the co-culture of CMs and endothelial cells. Kamei et al. [98] reported a soft lithography-based microfluidic device that uses integrated pneumatic valves and pumps to generate a precise fluid flow to simulate the side effects of cancer drugs. The system includes a set of cell culture chambers with a microfluidic system that simulates the blood circulation system and connects various tissue cells. Human healthy heart cells (hCMs) and liver cancer cells (HepG2) are co-cultured in separate chambers (Figure 3C). This device evaluates the side effects of doxorubicin on heart cells, which are caused by HepG2 cells delivering toxic metabolites to heart cells through circulation.

### 5.3. Brief Summary

Drug-induced cardiotoxicity is a critical issue in drug development. Unfortunately, current in vitro methods lack accuracy in the prediction, judgment, and treatment of cardiotoxicity. Development of microfluidic technology has made the heart chip a new type of in vitro tool, but there are still several core issues that need to be resolved, such as accurate real-time imaging which is difficult to achieve due to the high thickness of the chip and suitable biomarkers [99]. The current research focuses on solving these problems by integrating on-chip analytical techniques such as high-performance liquid chromatography, mass spectrometry, and electrophoresis [100]. Another challenge of the cardiac chip is to integrate different types of cells into a single tissue to better simulate the in vivo environment. However, due to the complexity of the in vivo environment and the long-term in vitro culture required for tissue stability, this can be a costly and challenging process. The cardiac chip enables integration of sensing devices, multi-cell co-culture, and 3D tissue formation. It is a new technology with rapid development and great potential, and it can become a new type of pre-clinical drug screening tool to evaluate drug cardiotoxicity.

## 6. Drug Neurotoxicity Testing by Nerve-on-a-Chip

Nerve-on-a-chip still is in its infancy. This section describes the research progress of neurochips, its application in toxicity detection, and research related to neurodegenerative diseases.

### 6.1. Research Progress of Neural Chips

Maher et al. [101] designed a set of microwell structures on a chip, which can achieve a one-to-one correspondence between neuron cells and metal microelectrodes. At the same time, each microwell has a hole through the center and pores around it. Synaptic connections can be established with peripheral neurons. In order to enhance the synaptic connection, Service et al. [102] designed a pattern on the surface of the chip, coated it with a thin layer of diethylenetriamine (DETA) that facilitates the growth of nerve cells. They successfully observed the directional growth of the synapses of the neuron. Maher et al. [101] also designed a chip in which microelectrodes were fabricated to stimulate and detect neuron cells at the same time, using a voltage-sensitive fluorescent dye whose excitation spectrum can change with the membrane potential.

Adhesion of neuron cells on the silicon surface is a key issue in the neural chip fabrication. Fan et al. [103] directly injected hydroxide ions into the silicon surface, thereby enhancing the hydrophilicity of the silicon surface and promoting cell adhesion. Qingjun Liu [104] integrated photo-addressable potential sensors on the neurochip, cultured olfactory sensory neurons on it, and fabricated a bioelectronic nose, which expanded the applications of the neural chip. Kang W et al. [105] integrated microchannels, cell culture chambers, built-in electrodes, and porous substrates in a neurochip that enabled long-term cell culture, differentiation, and transient transfection.

### 6.2. Application of Neural Chips in Toxicity Detection

Schmidt et al. [106] summarized that drug induced neurotoxicity occurs through six pathways, including the blood-brain barrier, lipid-rich structures, energy requirements, synaptic transmission, nerve cell structure, and neurobiochemistry, and toxicological testing methods should include test systems, exposure protocols, test endpoints, and prediction models.

Shichang Liu et al. [107] cultured rat cortical neurons on the surface of the microelectrode array fabricated using neural chip technology, and found that three nanomaterials (nano carbon black, nano Fe_2_O_3_, and nano TiO_2_) can produce neurotoxic effects by interfering with the electrical activity of neural networks. Nierode et al. [108] described high-throughput screening of human neural progenitor cells using a 3D cell culture chip. The microarray chip consisted of two complementary polystyrene chips containing 532 microwells for cell culture. The protein expression, viability, acute toxicity, and antiproliferative effects of 24 compounds were detected based on fluorescence analysis. Kafi et al. [109] grafted a nano-scale film of an arginine–glycine–aspartic acid tripeptide sequence on the surface of the chip for fixation of neurons and facilitated electrochemical measurement. They used this chip to monitor the toxic effects of quantum dots, graphene oxide, and cosmetic compounds.

**Figure 4:**
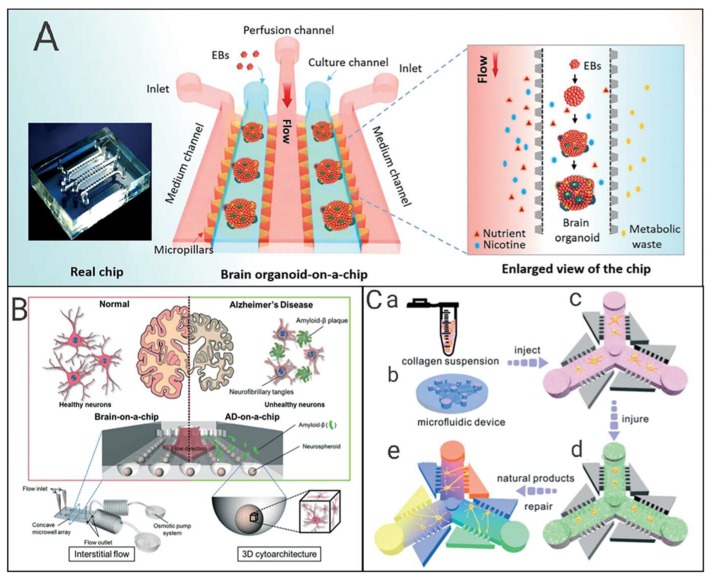
Schematic diagram of the research progress of neural chips. (**A**) The structure of the human brain organoid-on-a-chip to model prenatal nicotine exposure [112]. (**B**) Three-dimensional brain-on-a-chip with an interstitial level of flow and its application as an in vitro model of Alzheimer’s disease [114]. (**C**) A 3D microfluidic device to quantify orientational regeneration of injured neurons in AD by natural product concentration gradients [115].

Zhe Qu et al. [110] tested the toxic effects of nine selected drugs on Sprague Dawley (SD) rat neural stem cells using neurosphere technology. The size of neurospheres varied with toxic chemicals, and the results were consistent with the known neurotoxicity of these drugs. Xiannuo Zheng [111] developed a neurochip with a C-type dam structure, and realized long-term continuous observation and photographing of target cells. The toxicology of three different structures of quantum dots (CdTe, CdTe/CdS, and CdTe/CdS/ZnS) was studied on the chip platform in terms of the morphological changes, cell survival rate, and cell division and proliferation. Yaqing et al. [112] created a microfluidic chip system to culture human-induced pluripotent stem cell (hiPSCs) derived brain organoids in a controlled manner. The chip was composed of two parallel culture chambers and three independent culture medium channels. The two parallel culture chambers were separated by a central channel. The culture chamber was used for 3D culture and the formation of brain organoids. The central channel was used for perfusion. Apoptosis of brain organoids exposed to nicotine was studied on this platform (Figure 4A). Various biomarkers including TUJ1, SOX2, NESTIN, PAX6, PAX2, ISL1, CTIP2, and TBR1 were detected. Immunohistochemical staining and real-time quantitative PCR were performed. This brain organoid chip provided a universal and attractive platform for studying neurodevelopmental disorders in the early stages of pregnancy, and can be extended to brain disease research and drug testing applications. 

### 6.3. Neural Chips and Neurodegenerative Diseases

Alzheimer’s disease (AD) is a typical neurodegenerative disease. The accumulation of amyloid-beta (Aβ) is widely accepted as the cause [113]. Park et al. [114] designed and fabricated a concave microwell array in which neurospheres were cultured in dynamic conditions driven by osmotic micropump. The toxic effects of Aβ on nerve cells were measured using static and fluidic conditions. This 3D culture-based microfluidic chip is considered as an in vitro brain model for neurodegenerative diseases and high-throughput drug screening (Figure 4B).

Tang et al. [115] designed a microfluidic chip with an asymmetric side channel connected to the central channel by a series of microslots, and built a model of AD neuron damage using 6-OHDA. Three iridoid glycosides (catalpol, gardenoside and harpagide) were selected to investigate the regenerative effects on injured neurons, which have the potential to facilitate drug screening and optimization of treatment options for neurodegenerative diseases (Figure 4C).

Van de Wijdeven et al. [16] designed a microfluidic chip with open cell chambers and interconnected channels, which was flexible for the adjustment of the number and length of nodes, and direction of the channel, reducing flow induced shear stress on cell to construct a multi-node neural network.

### 6.4. Brief Summary

Drug-induced neurotoxicity is an essential issue in drug development. Unfortunately, current in vitro culture methods lack sensitivity and accuracy in the detection and judgment of neurotoxicity. The development of neural chip technology shows unparalleled advantages in neurotoxicity testing, highly simulating the complex physiological environment of human brain cells, and continuous perfusion can also achieve long-term dynamic culture. However, there is an urgent need to systematically combine in vitro, in vivo and clinical examinations to develop a series of biomarkers that can be safely and effectively detected in order to reliably monitor nerve damage and conduct in-depth research on repairing neurons. Neural chips are a new technology with rapid development and broad prospects that can become a new pre-clinical drug screening tool for evaluating neurotoxicity of drugs, which will definitely have a significant impact on the future survival and quality of life of humans.

## 7. Drug Toxicity Evaluation by Other Organ-on-Chips

### 7.1. Gut-on-a-Chip

The intestine is an essential site for drug metabolism, and most drugs undergo overall first-pass metabolism in it [116,117]. It is crucial to develop reliable in vitro models to study the intestinal metabolism of drugs involving multiple kinds of bacteria. This section will briefly introduce the current application of intestinal chips in drug toxicity testing.

The microfluidic device can provide laminar flow and control the volume of fluids, integrate TEER detection, and co-culture a variety of cells. These advantages allow gut-on-a-chip to overcome the shortcomings of traditional cell models and organoids. Most intestinal chips contain two hollow channels separated by an ECM-coated or polyester-coated polycarbonate porous membrane with immortalized human intestinal epithelial cells cultured on the membrane surface. Kim et al. cultured human intestinal epithelial (Caco-2) cells in a microdevice. Under the action of shear stress, the epithelial cells rapidly polarized, resulting in natural folds, which mimicked the structure of intestinal villi in vitro. Also, they confirmed that typical intestinal microorganisms (*Lactobacillus rhamnosus* GG) could be cultured in the device for more than a week, which is very similar to humans [118]. Subsequently, this group found that the intestinal villi in the device had particular cell and tissue functions, which were able to secrete mucus and had a robust drug metabolic activity for CYP3A4 [119]. In 2015, the Kim group cultured the human intestinal epithelial cells and some intestinal microorganisms in direct contact with the epithelial cells above the intestinal chip and analyzed the inflammatory mechanism caused by excessive growth of intestinal bacteria [120]. The series of studies above show the potential of intestinal chips in physiopathology and drug gut toxicity studies.

**Figure 5:**
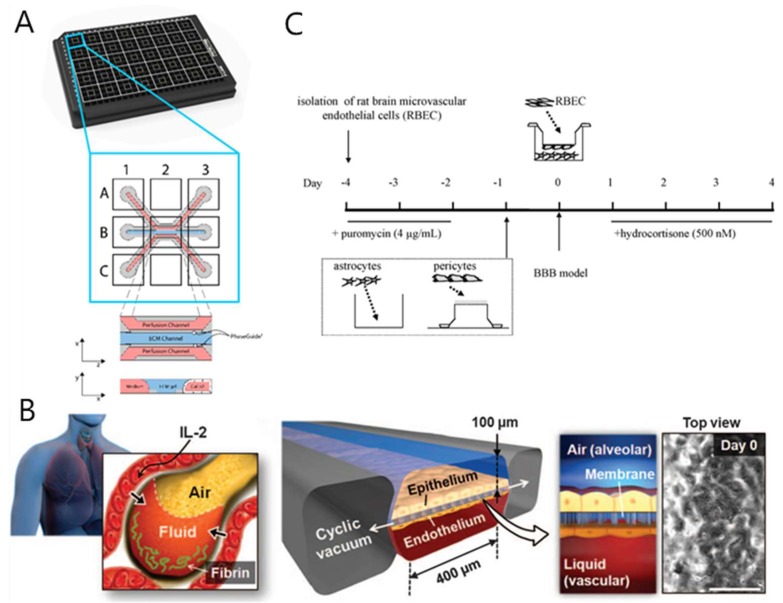
Some other-organ-on-a-chip systems for drug toxicity testing. (**A**) Schematic representation of the 3-lane OrganoPlate gut-on-a-chip model [122]. (**B**) A microengineered model of human pulmonary edema [127]. (**C**) Schematic drawing of the preparation of the in vitro blood–brain barrier (BBB) model [132].

Intestinal chips are often used to study drug metabolism and related diseases. Li’s team constructed an intestinal chip on which the intestinal epithelial cells formed a dense cell layer that showed higher alkaline phosphatase (ALP) and stress insensitive (SI) gene expression compared to traditional cell models [121]. They used the device to test the metabolic capacity of verapamil and isofluamide, and the results showed that intestinal cells had high levels of CYP450 enzyme expression, which enhanced the metabolism of oral drugs. Claudia et al. constructed a high-throughput intestinal chip device to mimic inflammatory bowel disease (IBD) in vitro (Figure 5A) [122]. Inhibition of crucial inflammation regulators RELA and MYD88 by on-chip adenovirus short hairpin RNA (shRNA) transduction can reduce the IBD phenotype by reducing cytokine production. These results also show the potential of gut-on-a-chip for both efficacy screening and drug toxicity testing.

A complete digestive system chip can be constructed based on the intestinal chip. Haan et al. [123] developed a small cell-free enzymatic digestion system with three microreactors in series to simulate the digestive functions of the mouth, stomach, and small intestine. The nutrients processed by the system are transferred to an intestinal absorption module, which is an intestinal chip containing human intestinal epithelial cells. This study provides new insights for in vitro studies of the digestive system.

### 7.2. Lung-on-a-Chip

During normal inhalation, the pressure in the pleura decreases, and air inhaled into the lungs causes the alveoli to expand, causing the endothelium to dilate the alveolar epithelium and adjacent capillaries. Many drugs can cause pulmonary toxicity, especially some chemotherapy drugs. Pulmonary toxicity can be divided into two categories based on the way lungs are exposed to the poison [124]. Drugs that disrupt the balance of the respiratory system are the major type of lung toxicity. At the same time, manufactured nanoparticles (NPs) that people come into contact with by inhalation can also cause toxic irritation [125]. This section will briefly introduce the application of lung-on-a-chip in studying lung toxicity and related diseases.

Dongeun Huh et al. reconstructed critical functional alveolar-capillary interface in vitro, and they set two lateral micro-chambers into a microfluidic device to simulate pressure-driven stretching of the lungs during breathing [126]. This design can simulate the dynamic mechanical deformation of the alveolar–capillary interface caused by respiratory motion. They also observed that when angiopoietin 1 (Ang-1) was co-administered with IL-2, the pulmonary epithelial cells were stable, and there was almost no vascular leakage caused by IL-2. This result indicates that Ang-1 prevents the formation of acellular gaps and that mechanical strain energy activates transient receptors or potential vanillin 4 (TRPV4) ion channels. This versatile system enables direct visualization and quantitative analysis of various biological processes in intact lung organs, which is impossible with animal models and traditional cell culture models. Later, the team built up a lung-on-a-chip system that can simulate lung function in normal and disease states (Figure 5B) [127], further expanding the application prospects of lung-on-a-chip technology in drug screening and toxicity testing. Zhang et al. designed a lung chip with a sandwich structure, which has three parallel channels. The device was separated by a matrix gel membrane in the middle, and endothelial cells and human alveolar cells were cultured in both channels. They used the device to evaluate the effects of titanium oxide (TiO2) and zinc oxide (ZnO) nanoparticles on cell morphology, connexin expression, reactive oxygen species (ROS) generation, and epithelial and endothelial cell apoptosis [125]. Yang’s group built a nanofiber membrane-supported lung chip and evaluated the toxicity of gefitinib, an anti-tumor drug targeting epidermal growth factor receptor (EGFR) [128]. Felder et al. developed a lung chip and performed a wound healing test on the chip to test the role and physiological circulation of human liver growth factor (rhHGF). The results proved that cyclic mechanical stretching had a significant effect on the wound healing process. The main results of these studies will help us to understand the complex pathogenesis of idiopathic pulmonary fibrosis (IPF) [129].

In summary, several studies conducted on the construction of lung chips obtained results that are more consistent with in vivo tests, demonstrating the potential of this model in studying respiratory disorders and drug screening.

### 7.3. Blood–brain Barrier (BBB)-on-a-Chip

There are still many microfluidic chips that mimic other human tissues or structures with the potential for drug screening and toxicity testing. This section will briefly introduce the research on BBB-on-a-chip.

The BBB is closely related to drug absorption and metabolism. At the level of brain microvasculature, BBB serves as a highly dynamic and functional interface between the systemic circulation and the central nervous system. Its function is to maintain a stable brain environment and protect the central nervous system. The BBB strictly and accurately regulates the transport of essential molecules and nutrients necessary for neuronal function. From a functional and structural point of view, BBB consists of highly differentiated vascular endothelial cells (VECs), which are arranged in the brain microvasculature [130]. In order to better study the relevant mechanisms of the BBB and the role of drugs through the blood–brain barrier in vitro, the researchers developed a BBB-on-a-chip model using the chip platform. Nicolazzo et al. [131] innovated a microfluidic in vitro model of the blood–brain barrier. Microfluidic blood–brain barrier (μBBB) were cultured with b.End3 endothelial cells, both with and without co-cultured C8-D1A astrocytes. Nakagawa et al. [132] proposed a new BBB model, in which primary rat brain endothelial cells, pericytes, and astrocytes were co-cultured (Figure 5C). Booth et al. [133] developed a μBBB with a dynamic environment of fluid shear stress and a relatively thin two-cell layer interface. The validity of the model was tested by TEER testing.

Vatine et al. used induced pluripotent stem cell (iPSCs) derived brain microvascular endothelial cells (iBMECs), astrocytes, and neurons to construct a complete human BBB chip. iBMECs form a tight monolayer that expresses cerebrovascular-specific markers [134]. The use of iPSCs from patients with neurological diseases can predict the lack of disease-specific transporters and the disruption of barrier integrity. By combining organ chip technology with human iPSC-derived tissue, the team created a neurovascular unit that can recombine complex blood–brain barrier functions, provide a platform for modeling heritable neurological diseases, and advance drug screening of personalized medicine.

### 7.4. Brief Summary

The design principle of the organ-on-a-chip system is to reconstruct the physiological functions of the organ system in vitro. Ideally, the microfluidic chip model can simulate the smallest functional unit of each organ system. In addition to the organs mentioned above, microfluidic chip models of skin [135], vasculature [136], and tumors [137] are also widely used in drug discovery and preclinical testing. With further technical development, more and more organs and tissues can be simulated on microfluidic chip models. Although there are still deficiencies in organ size and blood flow conversion [138], this technology has gradually matured.

## 8. Drug Toxicity Evaluation by Multi-Organ-on-a-Chip

### 8.1. Liver-Kidney-on-Chip

Chang et al. built a liver–kidney chip model to detect the renal toxicity of aristolochic acid on humans [139]. They demonstrated that human liver cell-specific metabolism of AA-1 increases its cytotoxicity to human renal proximal tubular epithelial cells, forms aristololactam adducts, and releases kidney injury biomarkers. AA-1 biotransformation into nephrotoxic metabolites through the liver involves nitro reduction followed by sulfate binding. The team determined that the sulfate conjugate of the aristololactam product of AA-1 (AL-1-NOSO3) produced by liver NQO1 in human tissue-based systems is the nephrotoxic form of AA-1. Theobald et al. developed a simple liver–kidney chip model in which liver cells stably grow and express related biomarkers [140]. They applied the system to study the biotransformation and toxicity of aflatoxin B1 (AFB1) and benzostyrene (BαP), and the interaction of these two drugs with other chemicals. The results show that in this model, the toxicity and metabolic response of drugs can be evaluated in a flow-controlled manner. Later, the team set up a multi-chamber liver-kidney chip to study drug metabolism in vitro [141]. The pump-driven vitamin D3-containing culture medium produced an eluate containing vitamin D3 metabolites through a microfluidic chip, where LC–MS/MS showed strong 25-hydroxyvitamin D accumulation.

### 8.2. Other Multi-Organs-on-Chips

Jong et al. developed a microfluidic device incorporating artificial liver, tumor, and bone marrow in which cells were cultured in hydrogels and microchannels and served as artificial blood vessels to test the cytotoxicity of anticancer drugs. The microfluidic network provides a platform for simulating the pharmacokinetics and pharmacodynamics of drugs in the human body, and the three-dimensional hydrogel provides a tissue environment more similar to physiological requirements [142]. The cytotoxic effect of Tegafur, an oral prodrug of 5-fluorouracil (5-FU), on each cell line, was tested using the micro cell culture analog (mCCA) and compared with a 96-well microtiter plate. The results showed that mCCA was able to reproduce the metabolism of Tegafur to 5-FU in the liver and consequent death of cells by 5-FU, while the 96-well microtiter plate did not. Oleaga et al. reported a multi-organ-on-a-chip including artificial heart, muscle, neuron, and liver, which could be maintained for 14 days. Computer simulation of the platform established the flow and provided the physiologically required shear stress [143]. After 48 h of exposure, the toxicity of five drugs in seven days was evaluated. The results obtained were generally consistent with the toxicity results already published from animal experiments and clinical testing. Li’s group proposed a new multi-layered organ chip device that can simultaneously assess drug metabolism and its efficacy and cytotoxicity in different organ-specific cells. Four cell lines representing liver, tumors (breast and lung cancer), and healthy tissues (stomach cells) were cultured in separate microcavities in multilayer microdevices [144]. From this study, it can be observed that the multi-organ-on-a-chip can reflect overall toxic effects of drug candidates on the whole body. McAleer et al. investigated the time-dependant pharmacokinetic/pharmacodynamic (PKPD) of tefenadine by a heart–liver system. The team used a metabolizable liver module to convert terfenadine to fexofenadine to regulate this response [145].

### 8.3. Brief Summary

Once the drugs enter the body, they penetrate the tissues through the blood circulation and reach various organs to exert therapeutic or toxic effects. The entire process involves interactions between many organs and tissues. The multi-organ-on-a-chip system can simulate the synergistic effects of multiple organs and build an organ network to study the absorption, distribution, metabolism, and elimination of drugs in the body [146]—for example, the liver–renal system and liver–intestine system mentioned above. Multi-organ chips still face particular challenges. For example, the culture conditions for each organ normally vary; however, on a multi-organ chip system, they normally are same. We need to differentiate the cell culture conditions on a multi-organ chip. With the rapid recognition of this issue, people began to discuss good cell culture practice (GCCP) [147] to establish cell quality standards and ensure the integrity and reproducibility of multi-organ chips in vitro. The future multi-organ-on-a-chip system will be more consistent and will be more applied in the fields of drug screening and toxicity detection.

## 9. Conclusions and Future Perspective

In preclinical drug development, using human cells to predict the toxicity of candidate compounds to improve the success rate of clinical trials is a topic that is being explored by almost all major pharmaceutical companies. The Food and Drug Administration (FDA) and drug regulatory agencies in other countries have already being focusing on organ-on-a-chip technology that has the potential to replace animal models in preclinical testing of drugs. Organ-on-a-chip technology can provide a good living environment for cells, tissues, or organs in vitro, and enhance its function and sensitivity to drug stimulation, which is very important in drug screening. Although the organ chips face some challenges in terms of materials, cellular origin, throughput, and validation, they have shown great potential for drug toxicity testing. A key goal of organ-on-a-chip technology in the field of toxicity is to enhance the reproducibility of the results and compare them with clinical data. If consistent results are obtained, this could eventually lead to a new drug toxicity testing paradigm.

## Figures and Tables

**Table 1:** Some common biomarkers for drug toxicity evaluation.

Toxicity	Biomarker	Reference	Specification	Whether Utilized with Organ Chips
Hepatotoxicity	ALT (alanine aminotransferase) AST (aspartate aminotransferase)	[19]	Diagnostic marker of liver damage	√
	ALP (alkalinephosphatase)	[19]	Diagnostic marker of cholestatic injury	√
	GLDH (glutamate dehydrogenase)	[20]	Early detection biomarker	×
	HMGB-1 (high-mobility group box 1)	[21]	Early detection biomarker	×
	K18 (keratin-18)	[22]	Early detection biomarker	√
	OCT (ornithine carbamoyltransferase)	[23]	Early detection biomarker	×
	GST-α (glutathione S-transferase α)	[24]	Early detection biomarker	√
	CYP (cytochrome P450)	[25]	Metabolic ability biomarke	√
	miRNA-122, miRNA-192	[26]	Genomic markers	√
	CDH-5 (cadherin-5)	[27]	Proteomics biomarker	√
	FABP1 (fatty acid binding protein 1)	[27]	Proteomics biomarker	×
nephrotoxicity	GFR (glomerular filtration rate)	[28]	Diagnostic marker of renal function	√
	SCr levels and urine output	[29]	Diagnostic marker of AKI (acute kidney injury)	√
	KIM-1 (kidney injury molecule-1)	[30]	Early detection biomarker	√
	NAG (N-acetyl-β-glucosaminidase)	[30]	Early detection biomarker	√
	NGAL (neutrophil gelatinase-associated lipocalin)	[31]	Early detection biomarker	√
	L-FABP (liver type fatty acid binding protein)	[32]	Early detection biomarker	×
	MCP-1 (monocyte chemotactic peptide-1)	[33]	Early detection biomarker	×
	CYs C (cystatin C)	[34]	Early detection biomarker	√
	OPN (osteopontin)	[35]	Early detection biomarker	×
	CLU (clusterin)	[36]	Early detection biomarker	×
	TFF3 (trefoil factor 3)	[37]	Early detection biomarker	×
	TEER (transendothelial resistance)	[38]	Biomarker of barrier functions	√
	miRNAs	[39]	Genomic markers	√
cardiotoxicity	LVDP (left ventricular formation pressure) LVSP (left ventricular systolic pressure)	[40]	Diagnostic marker of myocardial injury	√
	TnI (troponin I) TnT (troponin T)	[41]	Early detection biomarker	×
	BNP (brain natriuretic peptide) NT-proBNP	[42]	Early detection biomarker	×
	MPO (myeloperoxidase)	[43]	Biomarker of oxidative stress	×
	miR-146a, miR-1, miR-133, miR-208, miR-499	[44]	Genomic markers	√
	beating frequency, systolic stress, field potential	[45,46]	Mechanical markers	√
	TEER	[47]	Biomarker of barrier functions	√
neurotoxicity	plasma P-Tau (phosphorylated-Tau) T-Tau (total Tau)	[48]	Diagnostic marker of central nervous system (CNS) injury	×
	NF-H (neurofilaments heavy subunit)	[49]	Diagnostic marker of axonal injury	×
	miR-425-p, miR-21, miR-93, miR-191, miR-499,miR-328, miR-362-3p, miR-451, miR-486a	[50]	Genomic markers	√
	H-FABP (heart fatty acid binding protein)	[51]	Diagnostic marker of CNS injury	×
	SP, sCD40L, TIMP-1, MDA, CK-18	[52]	Early detection biomarkers	√
	MBG (marinobufagenin)	[53]	Biomarker of neuro-inflammation	×
other toxicities	Ghrelin	[54]	Biomarker of stomach/small intestine injury	×
	DAO (diamine oxidase) Citrulline	[54]	Biomarker of small intestine injury	×
	CD64, C-reactive protein	[54]	Biomarker of small/large intestine	√
	NOS isoenzymes	[55]	Breath biomarkers	√
	HO (heme oxygenase)	[56]	Biomarker of upper respiratory tract viral infections	√
	CYP (cytochrome P450) H2O2	[57]	Biomarker of pulmonary diseases	√
	Breath methylated hydrocarbons	[58]	Lipid peroxidation markers	×
	TEER	[59]	Biomarker of barrier functions	√
